# Effects of mother-offspring and father-offspring dynamics on emerging adults’ adjustment: The mediating role of emotion regulation

**DOI:** 10.1371/journal.pone.0212331

**Published:** 2019-02-13

**Authors:** Rebecca Y. M. Cheung, Man Chong Leung, Kevin K. S. Chan, Chun Bun Lam

**Affiliations:** 1 Department of Early Childhood Education, The Education University of Hong Kong, Hong Kong SAR, China; 2 Centre for Psychosocial Health, The Education University of Hong Kong, Hong Kong SAR, China; 3 Centre for Child and Family Science, The Education University of Hong Kong, Hong Kong SAR, China; 4 Department of Psychology, The Education University of Hong Kong, Hong Kong SAR, China; International Telematic University Uninettuno, ITALY

## Abstract

The present study tested a theoretical model of emotion regulation between parent-offspring dynamics and emerging adults’ adjustment. The mediating role of emotion regulation strategies, including cognitive reappraisal and expressive suppression, were investigated for the effects of mother-offspring and father-offspring dynamics on emerging adults’ adjustment. A sample of 352 Chinese emerging adults in Hong Kong (230 female, 121 male) participated in this study. Participants were asked to complete a set of self-reported questionnaires. Findings based on structural equation modeling indicated that greater mother-offspring intimacy and father-offspring intimacy predicted emerging adults’ better cognitive reappraisal and psychological, social, and general health. Greater mother-offspring conflict also predicted more expressive suppression and poorer psychological and social functioning. Distinctive mediation pathways as a function of parents’ gender were identified. These findings enrich the literature for parent-offspring dynamics and emotion regulation as explanatory processes of emerging adults’ adjustment.

## Introduction

The study of emotion regulation and well-being has received much scholarly attention over the last decade [1,2]. According to Thompson [3], emotion regulation is defined as “internal and external processes involved in initiating, maintaining, and modulating the occurrence, intensity, and expression of emotions.” Previous research suggested that emotion regulation changes from early adolescence to middle adulthood, with individuals’ use of adaptive emotion regulation strategies increasing with age [4]. Despite the variation of emotion regulation strategies in our lifetime, little is known about the precedents and correlates of emotion regulation in emerging adulthood. Emerging adulthood is a transition period from adolescence to adulthood, whereby individuals develop new values and identities [5]. Emerging adults encounter potential college or employment transitions involving academic, social, and geographical adjustment, struggles between family reliance and independence, and experiences of romantic love and responsibility. Although emerging adults may not rely on their families as frequently as they do from early childhood to adolescence, processes such as family cohesion, conflict, and expressivity continue to be important, as evidenced by their associations with coping behaviors and emerging adults’ adjustment in both Eastern and Western cultures [6,7,8,9,10].

According to the tripartite model of family influences on emotion regulation and adjustment [11], parent-offspring dynamics are pivotal to human development. In their model, Morris et al. [11] identified emotion regulation as a process through which family dynamics affect offspring’s adjustment. Indeed, the family emotional climate is closely intertwined with family members’ mutual emotional influences [12]. For example, family members may exchange their thoughts and feelings by responding to one another. Parents who frequently demonstrate hostile and hurtful emotions may model their dysregulated behaviors for their young children to imitate [13]. Such dynamics are salient from childhood to emerging adulthood, particularly in the Chinese context [14], where filial obligations and interpersonal harmony are highly valued [15,16]. Notably, common Chinese proverbs, such as 家和萬事興 (“All affairs prosper in harmonious families”), emphasize the importance of cultivating family harmony. Indeed, overt expressions of anger and conflict are regarded as shameful in diverse Asian contexts [17]. Disruptions of family harmony incur psychological costs, including greater depressive symptoms among Chinese individuals [18]. Consequently, adaptive family processes may be particularly salient to emerging adults’ functioning in the Chinese context.

Returning to the Western literature, parenting practice is a common family process associated with emerging adults’ emotion regulation and mental health. For example, Schwartz, Thigpen, and Montgomery [19] found that disapproving and dismissing parenting behaviors were cross-sectionally linked to emerging adults’ emotion dysregulation. Other cross-sectional studies similarly indicated that parents’ negativity, over-involvement, and psychological control were related to emerging adults’ greater emotion regulation difficulties and depressive symptoms, poorer psychological well-being, and lower life satisfaction [7,20,21]. Interestingly, from a cross-cultural perspective [22], Asian American emerging adults were less likely to retrospectively report on parent-child socialization of positive emotions and physical affection than were European Americans. In other words, despite the emphasis of family harmony [14,15], Asians reported fewer experiences of family socialization of positive emotions. These findings highlighted the similarities and differences in family dynamics between Eastern and Western cultures, particularly in relation to adjustment outcomes.

As for the association between family dynamics and health, longitudinal findings indicated that greater maternal support in mid-adolescence was associated with lower risk of cardiovascular disease at 28.7 years of age [23]. Other studies also demonstrated that positive processes including positive parenting behaviors, parental autonomy support, effective parent-offspring communication, family cohesion, and lower parent-offspring conflict were cross-sectionally related to lower levels of stress and depression and better adjustment among emerging adults [9,24,25,26,27]. In contrast, negative parent-offspring processes, as reflected by parent-offspring conflict, stress, and criticisms, cross-sectionally predicted risky levels of emerging adults’ distress, well-being, and academic achievements [28,29]. In the face of negative family dynamics, such as family violence and low family cohesion, emerging adults from both Eastern and Western cultures suffer from adjustment difficulties, both cross-sectionally [30,31] and longitudinally [32]. Supporting the tripartite model of family influences on emotion regulation and adjustment [11], converging evidence to-date has indicated that the parent-offspring relationship is crucial to well-being in emerging adulthood.

### Taking account of mother- vs. father-offspring dynamics on emerging adults’ adjustment

Both mothers’ and fathers’ dynamics and involvement are crucial to offspring’s adjustment over time [33,34]. Despite fathers’ contribution to their offspring’s development, attention in the literature mainly focuses on mothers’ characteristics in their emerging adult offspring cross-sectionally [20,35]. Emerging evidence accumulated to date has indicated that father-offspring relationship quality and closeness are also crucial to children’s development over time [35]. Specifically, both cross-sectional and longitudinal studies found that paternal emotion socialization practices, including supportive reactions, were associated with fewer mental health problems and better functioning among emerging adults [36,37,38]. Fathers and mothers also evidenced unique cross-sectional effects on the adjustment outcomes of their emerging adult offspring [39]. Given the complexity of family dynamics, delineating the effects of mother-offspring vs father-offspring relations on emerging adults’ psychosocial and health adjustment is central in family and developmental research.

### Emotion regulation and emerging adults’ adjustment

The use of adaptive vs. maladaptive emotion regulation strategies have implications for well-being [40]. Studies conducted in the last decade evidenced the relation between emerging adults’ emotion regulation and health outcomes [7,41,42]. Among the emotion regulation strategies researched in the field, such as rumination, acceptance, catastrophizing, and savoring [14,43,44], cognitive reappraisal and expressive suppression emerged as two widely-investigated strategies [2] associated with physical health, mental health, and social well-being [40,41]. Cognitive reappraisal refers to the process involving a reinterpretation of the meaning of an emotional stimulus and subsequently, leading to a change of the initial trajectory of an emotional response [45]. For example, family members’ anger and hostility associated with conflict may diminish upon their reinterpretations of the situation. Previous studies conducted in both Eastern and Western cultures suggested that cognitive reappraisal reduced negative emotions and heightened positive ones, thereby enhancing mental health and interpersonal functioning [1,40,46,47,48]. These findings highlighted the physiological, mental, and social benefits of cognitive reappraisal in emerging adulthood.

Contrary to cognitive reappraisal, expressive suppression involves hiding an emotional state by masking facial and bodily expressions. Although this strategy decreases the expression of negative emotional behavior, it potentially prevents the suppressors from experiencing positive emotions, increases experiences of negative emotions, and creates a sense of inauthenticity, which are detrimental to mental and physical health [40,49,50,51,52]. Suppression incurs social costs that involve reduced laughing, smiling, willingness to establish a friendship, and emotional disclosure [53]. It is also related to poorer social satisfaction, lower social support, and less closeness to others [54]. Consequently, compared to expressive suppression, cognitive reappraisal is a relatively more constructive way in modulating emotions due to its mental health benefits [2,55].

### Culture and emotion regulation

In a cross-sectional study on culture and emotion regulation, Matsumoto and colleagues [56] collected data from 23 countries and found that cognitive reappraisal and expressive suppression are both common in Chinese participants in Hong Kong. Unlike the other countries, these emotion regulation strategies yielded a very high correlation in Chinese participants (*r* = .90). Such an unusual correlation and their common use deserve marked attention in the Chinese context, particularly in relation to adjustment outcomes. Extending these findings, cross-sectional research further suggested that ethnicity moderated between expressive suppression and depressive symptoms, with a weaker association in Asian Americans than in European Americans [7]. Similarly, another study indicated that suppression was related to poorer psychological functioning in European Americans but not in Hong Kong Chinese. However, no ethnic differences were found in cognitive reappraisal [57]. More recent neuroimaging findings evidenced complex relations between expressive suppression, cognitive reappraisal, and emotions in a Chinese sample, in that frequent suppressors experienced difficulties in mitigating negative emotions through cognitive reappraisal [58]. Furthermore, although some research conducted with Chinese participants suggested health and psychological costs of expressive suppression [59], others suggested that psychological benefits of suppression, in that it reduced depressive experiences [60]. These mixed findings have created a gap in the literature concerning the psychological correlates of expressive suppression and cognitive reappraisal, which merits the present research investigation.

### The present study: Testing the mediating role of emotion regulation

**Fig 1:**
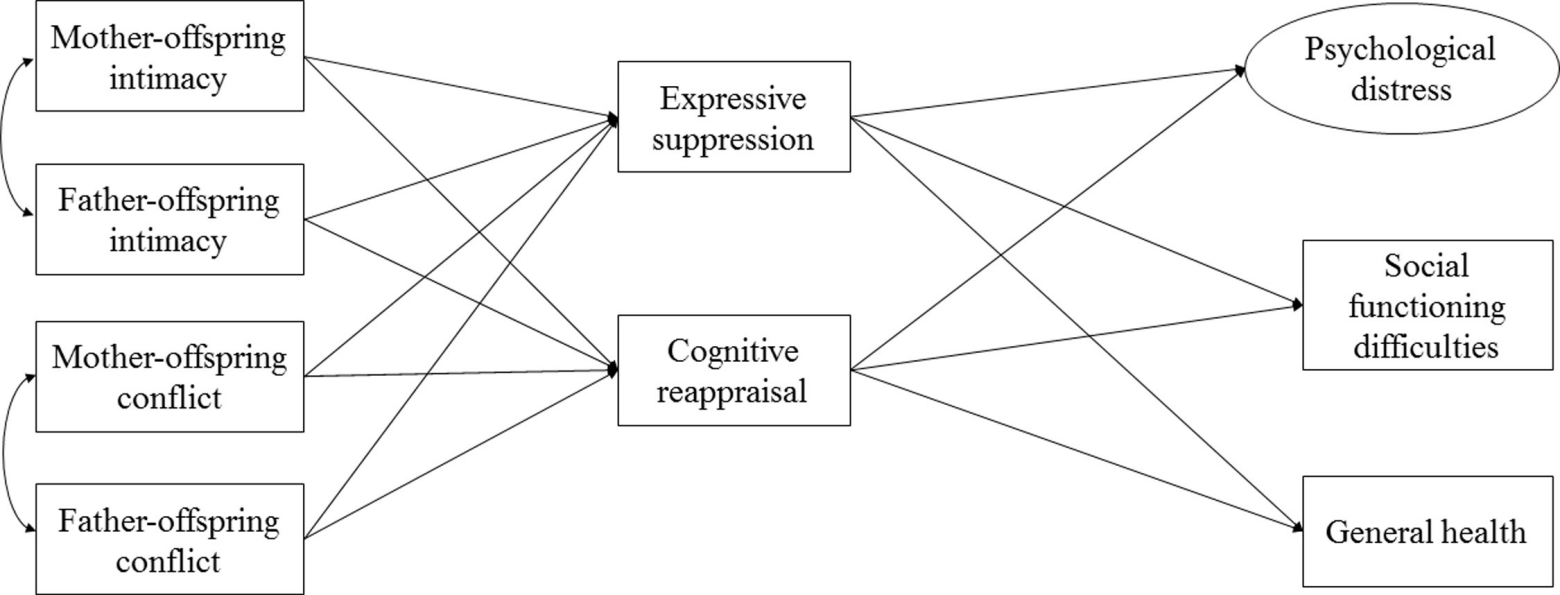
Conceptual model of emotion regulation between parent-offspring dynamics and emerging adults’ adjustment.

Extending the tripartite model of family effects on emotion regulation and adjustment to the Chinese context [11], the central goal of this study was to test the underlying mechanisms between parent-offspring dynamics and emerging adults’ adjustment in Hong Kong (see Fig 1). Specifically, we hypothesized that emotion regulation would mediate between parent-offspring dynamics and emerging adults’ adjustment. Given the cultural emphasis of harmony [15,16], we expected that disruptions in parent-offspring dynamics, regardless of parents’ gender, would be salient in predicting Chinese emerging adults’ poorer functioning. Despite the mixed findings between expressive expression and mental health outcomes in Chinese samples [7,57,60], in this study we expected that greater expressive suppression would undermine emerging adults’ overall adjustment. On the contrary, given the established positive relation between cognitive reappraisal and mental health in Asian samples [47,48], we expected that cognitive reappraisal would be linked to emerging adults’ better adjustment, as indexed by lower psychological distress, fewer social functioning difficulties, and better general health. Mother-offspring relationship and father-offspring relationship were each hypothesized to additively predict expressive suppression, cognitive reappraisal, and adjustment outcomes. Participants’ gender, age, family income, and number of siblings were added to predict the variables under study, as previous research suggested that these variables were crucial to understanding family dynamics and behavioral adjustment [61,62,63,64,65].

## Materials and methods

The study was approved by the Human Research Ethics Committee at The Education University of Hong Kong (Ref #: 2014-2015-0317). Written informed consent was obtained from all participants prior to the survey administration.

### Participants

Participants were 419 Chinese college students at a Chinese university in Hong Kong recruited through mass mailing. Of these participants, 67 (15.99%) came from single-parent families and were excluded from the analyses. The final sample consisted of 352 participants ranged in age from 18 to 27 years (*M* = 20.6; *SD* = 1.5), with 64.68% female (*n* = 271). Participants reported a median of having one sibling (*M* = 1.05; *SD* = 1.08) and a median of four members in the household (*M* = 3.82; *SD* = 1.03). The median monthly household income was HK$15000-$19999 (~US$1928.00-$2570.56). Most parents of the participants had completed high school education (71.71% mothers and 70.00% fathers). These sample demographics were comparable to the population demographics in Hong Kong [66,67]). Each participant received a supermarket coupon as a token of appreciation.

### Measures

#### Parent-offspring intimacy

Mother-offspring and father-offspring intimacy were measured by an 8-item parent-offspring intimacy measure adapted from a scale developed by Blyth, Hill, and Thiel [68]. In the original scale, Blyth et al. [68] used the 8 items to measure adolescents’ relationship with a “significant other” in their social world (e.g., a parent, a sibling, an extended-family member). Drawing from the adapted measure used in previous research [69,70], in this study the items concerning a “significant other” were similarly replaced with the participants’ “mother” and “father.” Participants rated on a 5-point scale from 1 (*not at all*) to 5 (*very much*) their intimacy with their mothers and fathers, respectively. Sample items included “How much do you share your inner feelings or secrets with your mother/father?” and “How much do you seek out your father/mother for advice/support?” In this study, Cronbach's alphas were .92 for both mother- and father-offspring intimacy.

#### Parent-offspring conflict

Parent-offspring conflict was assessed with a measure adapted from Smetana [71] on which participants rated the conflict frequency with each parent. In the original measure, Smetana [71] highlighted important domains in which parents and offspring had issues about emerging autonomy in adolescence. Drawing from the adapted measure used in previous research [72,73], in this study we assessed how often the emerging adults had conflict with their mothers and fathers in these domains. Participants rated on a 6-point scale from 1 (*not at all*) to 6 (*several times a day*) their frequency of having conflict over 10 life domains, such as chores, schoolwork, social life, romantic relationship, and money. Cronbach's alphas were .94 for mother-offspring conflict and .93 for father-offspring conflict.

#### Emotion regulation

The 10-item Emotion Regulation Questionnaire (ERQ; [40]) was used to measure emotion regulation strategies, including expressive suppression and cognitive reappraisal. Participants rated each item on a 7-point scale from 1 (*strongly disagree*) to 7 (*strongly agree*). The measure yielded adequate validity and reliability in previous research [40,74]. In this study, the ERQ demonstrated adequate internal consistency with Cronbach’s alpha = .73 for expressive suppression and .85 for cognitive reappraisal.

#### General health

The 5-item General Health Perceptions subscale of the Medical Outcome Study Short-Form Health Survey (SF-36; [75]) was used to measure participants’ perceptions of general health. Participants rated each item on a 5-point scale from 1 (*strongly disagree*) to 5 (*strongly agree*). The SF-36 yielded adequate validity and reliability in previous research [76]. In this study, the measure demonstrated adequate internal consistency with Cronbach’s alpha = .81.

#### Psychological distress

The 12-item General Health Questionnaire (GHQ-12; [77]) was used to measure participants’ psychological distress. Participants rated on a 4-point scale on three domains, including anxiety, loss of confidence, and social dysfunction [78]. The measure had adequate validity based on past research with early emerging adults [79]. In this study, the measure demonstrated adequate internal consistency with Cronbach’s alpha = .84.

#### Social functioning difficulties

The 8-item Social Functioning Questionnaire (SFQ; [80]) was used to assess participants’ social and relationship functioning difficulties. Participants rated each item on a 4-point scale. Sample items include “I have difficulties in getting and keeping close relationships” and “I get on well with my family and other relatives.” The measure yielded good validity and reliability based on previous research [80]. In this study, the measure demonstrated acceptable internal consistency with Cronbach’s alpha = .67.

## Results

Table 1 shows the correlations, means, and standard deviations for all study variables (see S1 Data). Mother-offspring and father-offspring intimacy were correlated at *r* = .38, *p* < .01. Similarly, mother-offspring and father-offspring conflict were correlated at *r* = .60, *p* < .01. Independent samples t-tests were conducted to examine potential gender differences on the outcome variables, as previous research indicated gender differences in psychological and interpersonal distress [81,82]. Compared to women, men reported more social functioning difficulties (*M*_*female*_ = 1.91, *SD*_*female*_ = .38; *M*_*male*_ = 2.07, *SD*_*male*_ = .40), *t*(416) = 3.81, *p* < .001. However, women and men reported similar levels of psychological distress and perceptions of general health, *p*s > .05. Age was significantly correlated with father-offspring intimacy (*r* = -.15, *p* < .01) but not the rest of the variables (*p*s > .05).

**Table 1:** Means, standard deviations, and correlations.

	(1)	(2)	(3)	(4)	(5)	(6)	(7)	(8)	(9)	(10)	(11)	(12)	(13)
(1) Sex	-												
(2) Age	-.09	-											
(3) Mother-offspring intimacy	.15^**^	-.10	-										
(4) Father-offspring intimacy	.06	-.12^*^	.38^***^	-									
(5) Mother-offspring conflict	-.05	-.00	-.07	-.00	-								
(6) Father-offspring conflict	-.09	.06	-.05	.11^*^	.66^***^	-							
(7) Expressive suppression	-.04	.05	-.10	-.01	.09	-.01	-						
(8) Cognitive reappraisal	.20^***^	-.04	.28^***^	.22^***^	-.05	-.08	.16^**^	-					
(9) Psychological distress—Anxiety	.02	.07	-.20^***^	-.10	.16^**^	.14^**^	.20^***^	-.10	-				
(10) Psychological distress— Social dysfunction	-.00	.05	-.10	-.09	.12^*^	.06	.14^**^	-.13^*^	.42^***^	-			
(11) Psychological distress— Loss of confidence	.05	.03	-.18^**^	-.11^*^	.18^**^	.10	.21^***^	-.17^**^	.63^***^	.45^***^	-		
(12) Social functioning difficulties	-18^**^	.08	-.39^***^	-.28^***^	.29^***^	.24^***^	.19^***^	-.26^***^	.45^***^	.26^***^	.49^***^	-	
(13) General health	.05	.01	.11^*^	.19^***^	-.08	-.03	-.19^***^	.10	-.32^***^	-.20^***^	-.24^***^	-.32^***^	-
*M*	-	20.49	3.45	2.93	2.43	2.12	4.27	4.87	2.07	2.02	1.86	1.94	3.34
*SD*	-	1.47	.81	.82	.99	.91	1.06	.79	.59	.38	.72	.38	.71

^*^p < .05

^**^p < .01

^***^p < .001

Structural equation modeling (SEM) was conducted using MPLUS (Version 7) [83] to examine the mediating role of emotion regulation between family processes and college students’ adjustment. Maximum likelihood method was used to examine the model fit to the observed covariance and variance matrices. A latent construct was created for psychological distress, with the subscales of anxiety, loss of confidence, and social dysfunction as indicators. Full information maximum likelihood estimation was used to handle missing data. Participants’ age, sex, family income, and number of siblings were incorporated in the structural model to control for the outcome variables. Bootstrapping was conducted to test the mediating effects, as it can yield more accurate estimates of the indirect effect standard errors than alternative approaches to testing mediation [84].

**Fig 2:**
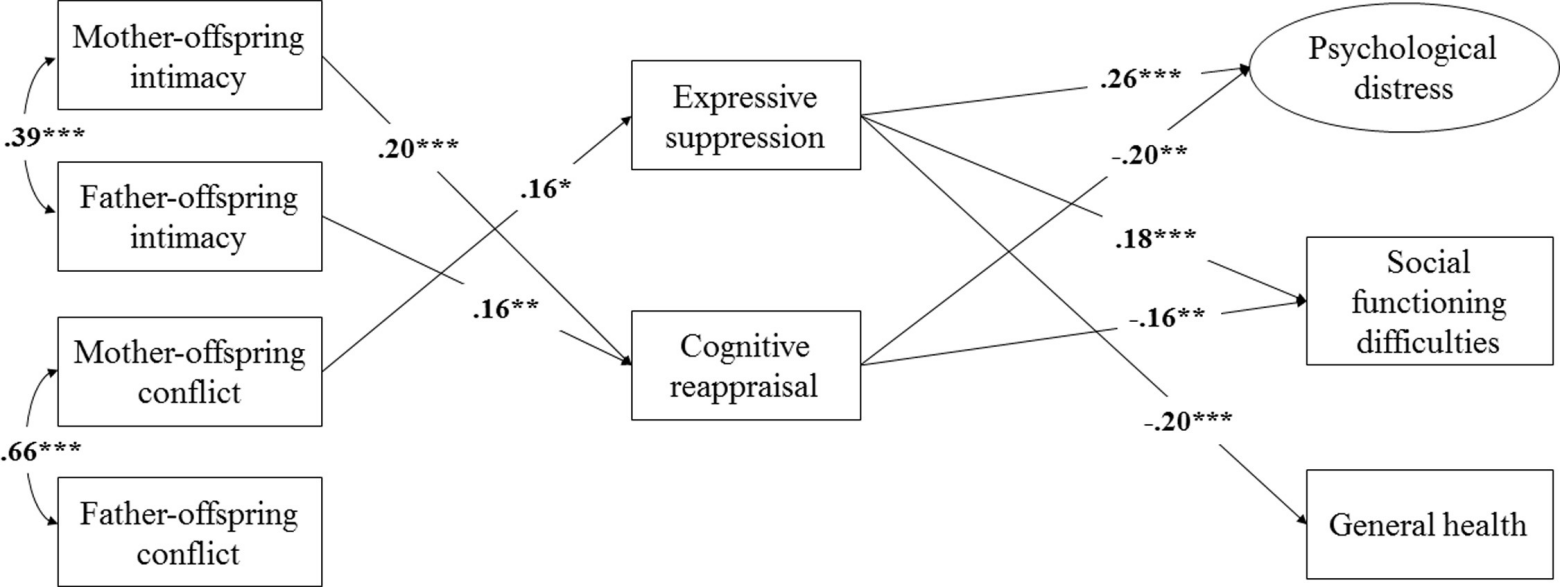
Mediation model of emotion regulation between parent-offspring dynamics and emerging adults’ adjustment.

As shown in Fig 2, the proposed model fit adequately to the data, χ^2^(40) = 51.87, *p* > .05, CFI = .98, TLI = .96, RMSEA = .03, SRMR = .03. Controlling for age, sex, family income, and number of siblings, mother-offspring intimacy predicted cognitive reappraisal (β = .19, *p* < .001), psychological distress (β = -.08, *p* < .05), and social functioning difficulties (β = -.11, *p* < .001). Father-offspring intimacy predicted cognitive reappraisal (β = .16, *p* < .01), social functioning difficulties (β = -.07, *p* < .01), and general health (β = .17, *p* < .01). Mother-offspring conflict predicted expressive suppression (β = .16, *p* < .05), psychological distress (β = .07, *p* < .05), and social functioning difficulties (β = .11, *p* < .001). Mother-offspring conflict was moderately related to father-offspring conflict (*r* = .59, *p* < .001). Similarly, mother-offspring intimacy was related to father-offspring intimacy (*r* = .26, *p* < .001). Table 2 indicates the unstandardized parameter estimates and standard errors in the model.

**Table 2:** Unstandardized parameter estimates, standard errors for the structural model.

Parameter	Unstandardized estimates (SE)
Measurement Model for Psychological Distress	
**➔** Anxiety problems	1.00 (.00)
** ➔ Social dysfunction**	.44 (.05)^***^
** ➔ Loss of Confidence**	1.30 (.11)^***^
Structural Model	
Mother-offspring intimacy	
**➔** Emotion suppression	-.12 (.08)
** ➔ Cognitive reappraisal**	.19 (.05)^***^
** ➔ Psychological distress**	-.08 (.04)^*^
** ➔ Social functioning difficulties**	-.11 (.02)^***^
**➔** General health	.00 (.05)
Mother-offspring conflict	
** ➔ Emotion suppression**	.16 (.08)^*^
**➔** Cognitive reappraisal	.03 (.05)
** ➔ Psychological distress**	.07 (.04)^*^
** ➔ Social functioning difficulties**	.06 (.02)^**^
**➔** General health	-.03 (.05)
Father-offspring intimacy	
**➔** Emotion suppression	.06 (.08)
** ➔ Cognitive reappraisal**	.16 (.05)^**^
**➔** Psychological distress	-.02 (.04)
** ➔ Social functioning difficulties**	-.07 (.02)^**^
** ➔ General health**	.14 (.05)^**^
Father-offspring conflict	
**➔➔** Emotion suppression	-.12 (.09)
**➔** Cognitive reappraisal	-.11 (.06)
**➔** Psychological distress	.02 (.04)
**➔** Social functioning difficulties	.05 (.03)
**➔** General health	-.001 (.06)
Emotion suppression	
** ➔ Psychological distress**	.11 (.03)^***^
** ➔ Social functioning difficulties**	.06 (.02)^***^
** ➔ General health**	-.13 (.04)^***^
Cognitive reappraisal	
** ➔ Psychological distress**	-.12 (.04)^**^
** ➔ Social functioning difficulties**	-.08 (.02)^**^
**➔** General health	.09 (.05)
Sex	
**➔** Emotion suppression	-.05 (.13)
** ➔ Cognitive reappraisal**	.20 (.09)^*^
**➔** Psychological distress	.09 (.06)
** ➔ Social functioning difficulties**	-.09 (.04)^*^
**➔** General health	.02 (.08)
Family Income	
**➔** Emotion suppression	.02 (.02)
**➔** Cognitive reappraisal	-.02 (.01)
**➔** Psychological distress	-.01 (.01)
**➔** Social functioning difficulties	-.01 (.01)
**➔** General health	-.002 (.01)
Number of Siblings	
**➔** Emotion suppression	.04 (.05)
**➔** Cognitive reappraisal	.03 (.04)
**➔** Psychological distress	.02 (.03)
**➔** Social functioning difficulties	.03 (.02)
**➔** General health	-.02 (.04)
Age	
**➔** Emotion suppression	.03 (.04)
**➔** Cognitive reappraisal	.01 (.03)
**➔** Psychological distress	.01 (.02)
**➔** Social functioning difficulties	-.002 (.01)
**➔** General health	.02 (.03)

^*^p < .05,

^**^p < .01,

^***^p < .001.

Bootstrapping was conducted in testing mediation effects. Specifically, the indirect effects of mother-offspring intimacy, father-offspring intimacy, and mother-offspring conflict on outcome variables including psychological distress, social functioning difficulties, and general health were examined (see Table 3). When the indirect effects (i.e., the product of regression coefficients), as represented here by confidence intervals, did not include zeros, we can infer that mediation occurred [84,85]. Using the current data, the 95% confidence interval [CI] based on 1000 bootstrap samples with replacement indicated that the indirect effects of mother-offspring intimacy on psychological distress and social functioning difficulties via cognitive reappraisal did not include a zero (95% CI: -.048, -.008; 95% CI: -.031, -.005, respectively), thereby indicating cognitive reappraisal as a mediator. Similarly, the 95% CI based on 1000 bootstrap samples with replacement indicated that the indirect effects of father-offspring intimacy on psychological distress and social functioning difficulties via cognitive reappraisal also did not include a 0 (95% CI: -.013, -.005; 95% CI: -.025, -.003, respectively), indicating cognitive reappraisal as a mediator. Finally, the 95% CI based on 1000 bootstrap samples with replacement indicated that the indirect effects of mother-offspring conflict on psychological distress, social functioning difficulties, and general health via expressive suppression did not include a 0 (95% CI: .001, .039; 95% CI: .001, .024; 95% CI: -.051, -.003, respectively), indicating expressive suppression as a mediator.

**Table 3:** Unstandardized parameter estimates and bootstrap analyses of specific indirect effects that do not include a zero.

Independent variable	Mediator variable	Dependent variable	Unstandardized indirect effect (SE)	95% CI indirect effect (lower, upper)
Mother-offspring intimacy	Cognitive reappraisal	Psychological distress	-.02 (.01)	-.048, -.008
	Cognitive reappraisal	Social functioning difficulties	-.02 (.01)	-.031, -.005
Mother-offspring conflict	Emotional suppression	Psychological distress	.02 (.01)	.001, .039
	Emotional suppression	Social functioning difficulties	.03 (.02)	.001, .024
	Emotional suppression	General health	-.02 (.01)	-.051, -.003
Father-offspring intimacy	Cognitive reappraisal	Psychological distress	-.02 (.01)	-.039, -.005
	Cognitive reappraisal	Social functioning difficulties	-.01 (.01)	-.025, -.003

## Discussion

Extending the tripartite model [11] to the Chinese context, the present findings demonstrated differential effects of parent-offspring dynamics on emerging adults’ adjustment via emotion regulation. Unique to this research was that both mother- and father-offspring dynamics were tested contemporaneously, such that we could compare and highlight distinctive findings as a function of parents’ gender. As indexed by supportive behaviors such as relationship satisfaction, understanding, and sharing of inner feelings [68], parent-offspring intimacy was identified to predict emerging adults’ emotion regulation and adjustment to psychosocial functioning and general health, regardless of parents’ gender. At the same time, mother-offspring conflict compromised emerging adults’ emotion regulation, mental health, and social functioning. In addition to advancing evidence for emotion regulation as an explanatory mechanism, these findings underscore the interplay between parent-offspring relationship and adjustment outcomes in emerging adulthood among Chinese individuals.

One of the key findings of this study was that expressive suppression mediated between mother-offspring dynamics and adjustment outcomes. Heightened mother-offspring intimacy and fewer instances of mother-offspring conflict were linked to lower expressive suppression, suggesting that constructive mother-offspring dynamics protected emerging adults from suppressing their emotions and other maladjusted outcomes. In other words, positive mother-child dynamics allowed offspring to be more emotionally forthcoming during the period of emerging adulthood. Heightened mother-offspring intimacy also was associated with greater cognitive reappraisal, psychological, and social adjustment. These findings were consistent with a previous study [86] conducted with a Chinese sample, in that maternal behaviors were linked to offspring’s cognitive reappraisal from 10 to 21 years of age. Supporting Morris and colleagues’ theoretical framework [11], emotion regulation served as a mechanism through which mother-offspring dynamics affected adjustment of emerging adult offspring.

Within the father-offspring dyad, cognitive reappraisal mediated between father-offspring intimacy and emerging adults’ mental health and social functioning. Surprisingly, although father-offspring conflict was moderately associated with mother-offspring conflict, father-offspring conflict did not predict emerging adults’ emotion regulation over and above other mother-offspring dynamics. Consistent with recent findings that adolescents’ emotion regulation was more closely linked to mothers’ than fathers’ emotion socialization [87], the present study suggested that mother-offspring conflict was more salient in predicting Chinese emerging adults’ emotion regulation, particularly in expressive suppression, and other adjustment outcomes.

Another unanticipated finding was that neither mother- nor father-offspring conflict was related to emerging adults’ cognitive reappraisal. The missing link might be due, in part, to the measure of parent-offspring conflict we used to assess conflict frequency and domains. To more thoroughly investigate the role of parent-offspring conflict, future research may further differentiate multiple facets of conflict, including severity and tactics, on emerging adults’ expressive suppression and cognitive appraisal. For example, previous studies suggested that maltreated offspring demonstrated fewer emotion regulation skills than did their non-maltreated counterparts [88]. That is, severe levels of parent-child aggression and conflict were linked to children’s dysregulated behavior. Another study indicated that parents’ conflict tactics, such as withdrawal, were linked to offspring’s emotion dysregulation [89]. As such, future research should evaluate multiple facets of conflict and emotion regulation to draw a more comprehensive conclusion. As an alternative speculation for the null findings, perhaps the participants underreported conflict frequency in order to preserve “face”, as family conflict is sometimes perceived as a source of shame and disharmony in the Asian context [90,91]. To increase scientific rigor of research in this area, future research should, again, utilize different methods including observations, surveys, and vignettes to more accurately capture parent-offspring conflict.

The present study represented one of the first attempts to investigate emotion regulation strategies between mother- and father-offspring correlates and emerging adults’ adjustment in a Chinese context. Nevertheless, findings must be interpreted in light of their limitations. First of all, the cross-sectional design precluded us from inferring direction of effects and causality. Future scholars should investigate the variables longitudinally to minimize potential biases and establish their temporal sequence [92]. Second, the variables were assessed through self-report. Future studies should recruit multiple reporters, including mother-, father-, and sibling-reports, and utilize multiple methods of assessment. Third, previous research indicated that family processes and well-being were associated as a function of gender [93,94,95]. Although the role of offspring’s gender was not the focus of this study, gender might have moderated the associations between the variables. Future studies should delineate potential differences or similarities in the associations as a function of both parents’ and offspring’s gender. Fourth, we did not solicit the information concerning parent-offspring relationship status (e.g., biological parents, step-parents, or foster parents), which may contribute to the current findings. Previous research suggested that parent-offspring relationship status is crucial to family dynamics and offspring’s development [96,97]. As such, future studies should investigate how parent-offspring relationships may further explain the present findings. Finally, although cognitive reappraisal and expressive suppression are two common emotion regulation strategies [2], the investigation of other strategies may contribute to a more comprehensive examination of emotion regulation.

## Conclusion

This study calls attention to the relation between parent-offspring relationship and Chinese emerging adults’ adjustment. Findings highlighted distinctive effects of mother-child and father-child relationships on emerging adults’ emotion regulation, psychosocial adjustment, and general health. These findings add to the growing literature that delineates processes underlying adjustment outcomes in emerging adulthood [5]. Informed by the current findings, policy makers, researchers, and practitioners should carry forward translational research programs that enhance parent-offspring communications and emotion regulation in late adolescence and emerging adulthood. Longitudinal research and family interventions geared toward enhancing parenting strategies, emotion regulation, and adjustment in emerging adulthood merit future investigation.

## Supporting information

S1 Data Contains the data of the variables under study. (CSV)
